# NF-*κ*B, JNK, and TLR Signaling Pathways in Hepatocarcinogenesis

**DOI:** 10.1155/2010/367694

**Published:** 2010-11-28

**Authors:** Shin Maeda

**Affiliations:** Department of Gastroenterology, Yokohama City University, 3-9 Fukuura, Kanazawa-ku, Yokohama 236-0004, Japan

## Abstract

Hepatocellular carcinoma (HCC) is the third largest cause of cancer deaths worldwide. The role of molecular changes in HCC have been used to identify prognostic markers and chemopreventive or therapeutic targets. It seems that toll-like receptors (TLRs) as well as the nuclear factor (NF)-*κ*B, and JNK pathways are critical regulators for the production of the cytokines associated with tumor promotion. The cross-talk between an inflammatory cell and a neoplastic cell, which is instigated by the activation of NF-*κ*B and JNKs, is critical for tumor organization. JNKs also regulate cell proliferation and act as oncogenes, making them the main tumor-promoting protein kinases. TLRs play roles in cytokine and hepatomitogen expression mainly in myeloid cells and may promote liver tumorigenesis. A better understanding of these signaling pathways in the liver will help us understand the mechanism of hepatocarcinogenesis and provide a new therapeutic target for HCC.

## 1. Introduction

Hepatocellular carcinoma (HCC) is the third largest cause of cancer deaths worldwide, particularly in Africa and Eastern Asia. Major HCC risk factors include infection with hepatitis B (HBV) or C viruses (HCV) and cirrhosis associated with chronic inflammation. The fungal contaminant aflatoxin B_1_ and environmental pollutants, such as aromatic amines, vinyl chloride, polycyclic aromatic hydrocarbons, and nitrosamines are also risk factors [1]. 

The role of molecular changes in the acquisition of resistance or susceptibility to HCC and the importance of genetically susceptible and resistant murine models have been used to identify prognostic markers and chemopreventive or therapeutic targets. Studies with genetically modified mice have revealed many genes associated with HCC. Among them, we focus on toll-like receptors (TLRs) as well as the nuclear factor (NF)-*κ*B and the JNK pathways in HCC development in this paper.

## 2. Putative Mechanisms of HCC Development

Although many putative causal factors are involved in the development of HCC, carcinogenic mechanisms have been difficult to elucidate. Among them, particular HBV or HCV proteins induce HCC without other oncogenic alterations in mouse models [2]. It is thought that chronic liver injury resulting in compensatory proliferation of differentiated hepatocytes is one of the major pathogenic mechanisms underlying HCC development [3, 4]. Several signal transduction pathways such as NF-*κ*B and JNK are involved in the pathogenesis of these viruses. For example, the HCV core and HBx proteins are the most potent signal inducers for NF-*κ*B and AP-1 [5]. 

Another important etiology of HCC development is obesity-induced hepatosteatosis, which includes more severe complications such as nonalcoholic steatohepatitis (NASH), classified as nonalcoholic fatty liver disease (NAFLD) [6]. Several mechanisms have been proposed to explain how NASH increases HCC risk, including the prevalence of insulin resistance among obese individuals, which results in elevated circulating concentrations of insulin and insulin-like growth factor 1 (IGF-1) [7] and a low-grade inflammatory response with elevated production of cytokines, such as TNF and IL-6 [8]. In these cases, it seems that the NF-*κ*B and JNK pathways are critical regulators for the production of the cytokines associated with tumor promotion.

## 3. NF-*κ*B and HCC

### 3.1. NF-*κ*B Signaling Pathway

**Figure 1:**
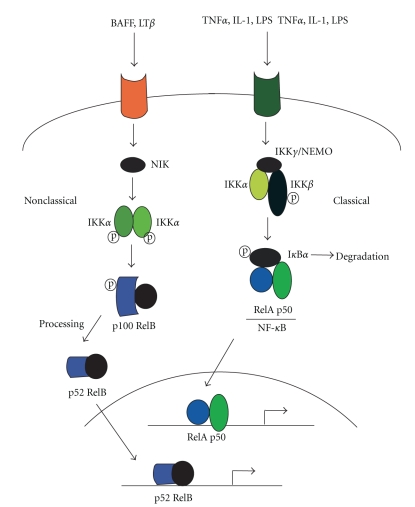


NF-*κ*B transcription factors are key regulators of innate and adaptive immune responses, inflammation, and cell survival [9]. Many proinflammatory stimuli activate NF-*κ*B, mainly via I*κ*B kinase-(IKK) dependent phosphorylation and degradation of the *κ*B inhibitor (I*κ*B) proteins. Five members, p65 (RelA), p50/NF-*κ*B1, c-Rel, RelB, and p52/NF-*κ*B2, belong to the mammalian NF-*κ*B family and are assembled by dimerization. Once activated, NF-*κ*B dimers stimulate transcription of genes encoding cytokines and antiapoptotic factors [10]. IKK consists of two catalytic subunits, IKK*α* and IKK*β*, and a regulatory component, NEMO/IKK*γ*. IKK activation occurs primarily through IKK*β* [10], whose absence increases susceptibility to tumor necrosis factor-*α*-(TNF*α*-) induced apoptosis [11] (Figure 1). 

In the nonclassical pathway, NF-*κ*B activation is triggered by members of the TNF family, including B-cell-activating factor, which belongs to the TNF family (BAFF), the CD40 ligand, and lymphotoxin *β*/LT*β*. Dimerized IKK*α* is activated by NF-*κ*B-inducing kinase (NIK), after which the p100 proteins are processed. After processing p100, p52/NF-*κ*B2 complexes move into the nucleus, resulting in the expression of genes that activate the adaptive immune response and the development of secondary lymphoid organs [12] (Figure 1).

### 3.2. NF-*κ*B Activation and Carcinogenesis

Tumor initiation means cellular immortality, which happens through DNA mutation, but the relationship with NF-*κ*B activation has not been considered in detail for this process. However, the first clue linking NF-*κ*B to cancer was recognizing that c-*rel*, which is a v-*rel* oncogene cellular homologue, encodes an NF-*κ*B subunit and that all of these proteins share the Rel homology DNA-binding domain [13]. Not surprisingly, overexpression of normal Rel proteins promotes oncogenic transformation.

Participation of NF-*κ*B activation in the carcinogenic promotion and progression stages has become clear in recent years. This promotion stage is the cell proliferative stage, and proliferation, antiapoptosis, angiogenesis, invasion, and metastasis become important [14].

It is unclear whether NF-*κ*B activation is directly associated with tumor cell proliferation. There are a few reports of a strong participation in transcriptional regulation of the expression of growth factors through NF-*κ*B activation. However, for the activation and infiltration of inflammatory cells, inhibition of NF-*κ*B activation may reduce expression of these growth factors. TNF*α*, which is a strong NF-*κ*B-activating factor whose expression is regulated by NF-*κ*B, is produced by macrophages due to inflammation and plays a central role in inflammation but has also been suggested as an accelerator factor of cell proliferation [15]. In addition, the possibility that NF-*κ*B activation operates on cell growth through cyclin expression is indicated in certain cell types [16]. In contrast, *Ikk*β** deletion accelerates hepatocyte growth. *Ikk*β**-deficient hepatocyte proliferative responses show heightened sensitivity to TGF-*α* [17], suggesting that involvement of NF-*κ*B activation in proliferation differs among cell types.

Antiapoptosis is also important for maintaining cancer cells. A large number of antiapoptotic factors, such as cIAPs, c-FLIP, and BclX, are controlled by NF-*κ*B activation [18]. Tumor cells with constitutive NF-*κ*B activation are highly resistant to anticancer drugs, and the inhibition of NF-*κ*B activity in those cells increases tumor sensitivity to these therapies [19–22]. 

Invasion and metastasis are pivotal processes for prognosis. Matrix metalloproteinases (MMPs) are produced by inflammatory cells and tumor cells and are key players in the degradation of the extracellular matrix and basement membranes; thus, they are important in tumor invasion. Gelatinases (MMP-2 and MMP-9), in particular, are prognostic factors in many solid tumors, and their expression is regulated by NF-*κ*B activation. The clinical application of an MMP inhibitor aimed at antimetastasis is expected [23]. Recent observations suggest that IKK*β*/NF-*κ*B activation controls the development of liver metastasis through IL-6 expression, which is associated with tumor cell proliferation and angiogenesis [24]. 

It is likely that NF-*κ*B activation is most critical in cancers in which inflammation acts as a tumor promoter, but a more general role for NF-*κ*B remains to be established for the inflammation mediated by its activation in cancers that are not associated with inflammation.

### 3.3. NF-*κ*B Activation and HCC

In clinical cases, constitutive NF-*κ*B activation has been frequently observed in tumor tissues rather than in nontumor tissues [25]. High expression of IKK*α* and IKK*β*, which are critical kinases for NF-*κ*B activation, is necessary to produce the malignant properties of liver cancer [26]. 

Although IKK*β*-deficient mice die at mid-gestation from uncontrolled liver apoptosis [27], the IKK*β* deficiency in the hepatocytes does not influence their function and growth [28]. However, following an injection of concanavalin A (ConA), liver damage becomes worse in IKK*β* hepatocyte-specific IKK*β* knockout mice, and TNF*α*, which causes ConA expression on the T cell surface, is responsible for the apoptotic action. This result suggests that IKK*β*/NF-*κ*B activation of hepatocytes is important for the survival of TNF*α*-mediated acute hepatitis and that inactivating NF-*κ*B may injure hepatocytes [28]. 

The roles of IKK*β* in carcinogenesis have been examined using diethylnitrosamine (DEN) as a chemical carcinogen. DEN does not require any assistance from inflammation-inducing tumor promoters if it is given to 2-week-old male mice. An increase in hepatocyte death coincident with an increase in reactive oxygen species (ROS) was observed in knockout mice when the acute reaction was analyzed following chemical carcinogen administration. Furthermore, cell death was accompanied by inflammation, and elevated hepatocyte death enhanced compensatory proliferation due to the strong regenerative capacity of the liver [29]. 

Decreased NF-*κ*B activity and elevated JNK activity promote TNF-*α*-induced cell death [30]. Accordingly, hepatocyte IKK*β* ablation results in higher DEN-induced JNK activity and increased cell death. Enhanced JNK activation in the absence of IKK*β* is dependent on ROS accumulation. Interestingly, a reduction in ROS production by the antioxidant BHA controls liver cell death and proliferation and reduces carcinogenesis [29]. 

Compensatory proliferation depends on the production of factors such as TNF-*α*, IL-6, and hepatocyte growth factor by nonparenchymal cells [31], and NF-*κ*B activation is important for producing these cytokines. Accordingly, hepatocytes and blood cells, including Kupffer cells, which are liver macrophages, were used in a carcinogenesis model of IKK*β* knockout mice, and the occurrence of HCC was reduced. These results suggest that IKK/NF-*κ*B activation in myeloid cells is important for producing liver growth factors [29].

In fact, DEN-induced hepatocarcinogenesis has been examined in IL-6 knockout mice and wild-type controls, and a marked reduction in HCC incidence was seen in IL-6 knockout mice. Interestingly, gender disparity is observed in mice given DEN, and ablation of IL-6 abolishes this gender difference [32]. 

Recently, IKK*β* deletion was observed to accelerate HCC development and enhance tumor cell proliferation. These effects of IKK*β* were correlated with increased ROS accumulation, which led to JNK and STAT3 activation. Accordingly, hepatocyte-specific STAT3 ablation prevents HCC development. These results suggest that the negative crosstalk between IKK*β* and STAT3 is a critical regulator in HCC development [33].

The IKK subunit NEMO/IKK*γ* is essential for activating the NF-*κ*B transcription factor, which regulates cellular responses to inflammation. Hepatocyte-specific ablation of NEMO causes the spontaneous development of HCC in mice. Tumor development is preceded by chronic liver disease such as NASH. These results reveal that NEMO-mediated NF-*κ*B activation in hepatocytes plays an essential physiological role to prevent the spontaneous development of NASH and HCC [34]. However, viral-induced activation of IRF3 and IRF7 depends on NEMO, suggesting that NEMO acts as an adaptor protein allowing RIG-I to activate not only NF-*κ*B, but also IRF signaling pathways. Ablation of NEMO in the liver may influence the IRF pathway [35].

### 3.4. NF-*κ*B and Therapeutic Target for HCC

Applying NF-*κ*B inhibitors for use as anticancer agents is expected. Through an antiapoptosis effect of NF-*κ*B, the inhibition leads cells to apoptosis. Furthermore, constitutive NF-*κ*B activation is observed in many cancer cells, and connection with a malignancy of the cancer is assumed. IKKs or I*κ*Bs are now the main targets of NF-*κ*B inhibitors. Many researchers have discovered various inhibitors that attenuate IKK*α*, IKK*β*, IKK*γ*, or I*κ*B*α*, and some are effective in animal models. In our group, NEMO-binding domain peptide (NBD), one of the inhibitors of IKK*β*, has shown chemopreventive effects in a mouse colitis model [36]. Discovering drugs that target these molecules appears to be attracting greater attention, and the development of specific IKK*β* inhibitors has progressed rapidly. Several novel, small-molecule IKK*β* inhibitors have demonstrated anti-inflammatory activity, and the advancement of IKK*β* inhibitors into clinical development is anticipated in the near future [37].

## 4. JNK and HCC

### 4.1. JNK Signaling Pathway

**Figure 2:**
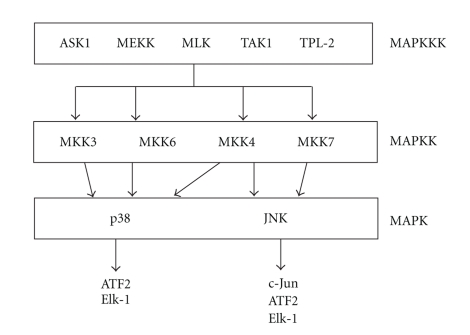


The c-Jun NH_2_-terminal kinase (JNK) belongs to a family of mitogen-activated kinases (MAPKs), together with extracellular regulated kinases (ERKs) and p38. The JNK subgroup of MAPKs is encoded by three loci; *Jnk1* and *Jnk2* are ubiquitously expressed, and *Jnk3* is expressed primarily in heart, testis, and brain [38–40]. JNKs are activated by stress signals and proinflammatory stimuli, and their activity increases following phosphorylation by the MAPK kinases, MKK4, and MKK7 [41] (Figure 2).

Immunoblot analyses suggest that a 46-kDa molecule represents JNK1 and a 54-kDa molecule represents JNK2. However, a recent analysis revealed that the 46- and 54-kDa molecules represent two isoforms of JNK1 and JNK2, whereas the 46-kDa JNK1 and 54-kDa JNK2 are dominantly expressed in most cell and tissue types [42]. Activated JNKs phosphorylate c-Jun, JunD, ATF, and other transcriptional factors, which are involved in the formation and activation of the AP-1 complex [43]. In addition, JNKs also phosphorylate other proteins that induce apoptosis, cell proliferation, or transformation, depending on the cell type and stimuli [44].

Using gene knockout mice, it has been shown that JNKs are involved in many processes, including liver inflammation and proliferation, neuronal apoptosis, T-cell activation, and insulin resistance [45–49].

### 4.2. JNK Activation and Liver Disease

In the liver, activating JNK was thought to be important for proliferation and apoptosis. In a knockout-mice analysis, JNK1 was associated with increased apoptosis in ConA-induced acute hepatitis [28]. Acetaminophen-induced liver injury is also JNK dependent [50, 51]. Furthermore, mice lacking both JNK1 and 2 expression in hepatocytes exhibit the same degree of injury in the development of hepatitis as control mice do, whereas mice without JNK1/2 in the hematopoietic compartment exhibit a profound defect in hepatitis that is associated with a markedly reduced expression of TNF-*α*. It is suggested that a role for JNK in the development of hepatitis, but identified hematopoietic cells as the site of the essential function of JNK [52]. JNK plays a pivotal role in the development of metabolic syndrome including NAFLD [48, 53]. Hepatic steatosis, inflammation, and fibrosis have been examined in mice fed a choline-deficient L-amino-acid-defined diet. The results showed less hepatic inflammation and less liver fibrosis despite a similar level of hepatic steatosis in JNK1-deficient mice compared with wild type, suggesting that JNK1 may be associated with the induction of diet-induced steatohepatitis and liver fibrosis [54].

### 4.3. JNK and Carcinogenesis

Tong et al. reported that JNK1 knockout mice spontaneously develop intestinal tumors, suggesting a role for JNK1 in suppressing intestinal tumor formation [55]. In contrast, mice lacking JNK1 were much less susceptible to N-methyl-N-nitrosourea-induced gastric carcinogenesis, which was correlated with decreased tumor initiation and diminished cell proliferation [56]. These findings suggest that the JNKs have tumor-promoting or tumor-suppressing functions, depending on the cell type or organ.

Tumor initiation indicates cellular immortality, which occurs due to a DNA mutation, and the relationship with the JNK activation is considered important. The presence of mutated ras genes in 30% of all human cancers suggests an important contribution to the development of human cancers. Overexpression of mutated ras genes causes transformation of a variety of rodent fibroblast and epithelial cell lines. Ras causes activation of a Raf-independent MAPK cascade, which leads to JNK activation [57], and inhibition of JNK activation also inhibits Ras transformation in NIH3T3 cells [58]. JNK1-dependent c-Myc and p21 are responsible for the diminished checkpoint function in tumorigenic hepatocytes [59].

JNK function is critical in the carcinogenic promotion and progression stages, as JNK phosphorylates a variety of genes associated with carcinogenesis. The promotion stage is the proliferative stage of the cell, which becomes immortal, and proliferation, anti-apoptosis, angiogenesis, invasion, and metastasis are important [14]. 

Growth factors activate receptor tyrosine kinases, and phosphorylated receptors transmit the signals through JNKs [60]. There is also participation in the transcriptional regulation of growth factors such as EGF through JNK activation [61]. Numerous studies have considered the proliferative effect following JNK activation. For example, in a liver regeneration mouse model, the number of Ki67-positive proliferating hepatocytes in *Jnk1^−/−^* mice was reduced by 80% compared with that in controls at 48 hours after a partial hepatectomy [59]. In an HCC model induced by DEN, several important genes associated with proliferation such as PCNA, cyclin D, and CDKs increased [4].

The expression of several angiogenic factors is also regulated by JNK. Vascular endothelial growth factor (VEGF) promotes proliferation and migration of endothelium cells. VEGF expression is also controlled by JNK activation [62]. Chemokines such as interleukin (IL)-8 are factors controlling leukocytes at the time of inflammation, but they function as blood vessel growth factors in tumor tissue [63, 64].

### 4.4. JNK Activation and HCC

JNKs are protein kinases, and many molecules are phosphorylated by JNKs. Approximately, 70% of HCC tissues, but not background tissues, show positive immunostaining for phosphorylated JNK, suggesting that JNK is frequently active in human HCC [65]. *In vitro* studies suggest that several HBV and HCV proteins are common etiologic pathogens for HCC-activated JNK in cancer cells [66]. 

In addition to the clinical samples and *in vitro* studies, JNK activation has been critically important in *in vivo* mouse models. In hepatocyte-specific *Ikk*β**-deficient mice, DEN-induced liver cancer increased and JNK activation was elevated simultaneously [29]. To determine the role of JNK in these models, JNK1-deficient mice were interbred with *Ikk*β**-deficient mice, and the results showed that JNK1 is a critical factor for the increased incidence of HCC in *Ikk*β**-deficient mice [4]. Additionally, to determine the role of JNK1 in DEN-induced hepatocarcinogenesis, wild-type and *Jnk1*-deficient mice were injected with DEN. All mice given DEN developed typical HCCs, but the number of detectable HCCs was reduced fivefold by the JNK1 deficiency. Thus, JNK1 is required for efficient HCC induction in response to DEN administration [4]. 

The downstream molecules phosphorylated by JNK have been extensively investigated. Originally, JNKs were identified as protein kinases that phosphorylated c-Jun on serine residues [67]. c-Jun is a well-characterized oncogene, especially in liver [68], and its phosphorylation by JNK may be relevant in HCC development. The AP-1 transcription factors, c-Jun and c-Fos, are composed of homo- and heterodimers with basic region-leucine zipper proteins belonging to the Jun (c-Jun, JunB, and JunD) and Fos (c-Fos, FosB, Fra-1, and Fra-2) subfamilies, all of which recognize the AP-1 binding site or TPA-response element in the regulatory region of AP-1 target genes [69]. Liver carcinogenesis induced by DEN is dramatically reduced in mice lacking c-Jun in hepatocytes. A putative mechanism accounting for the reduced tumorigenic effect was prevention of apoptosis by c-Jun, and the prevention of apoptosis was associated with p53 [68]. 

The tumorigenic effect of JNK1 in the liver is mediated through positive gene regulation or by cell proliferation molecules such as cyclins and CDKs and metastatic factors such as MMPs, VEGF, and others [4]. 

**Figure 3:**
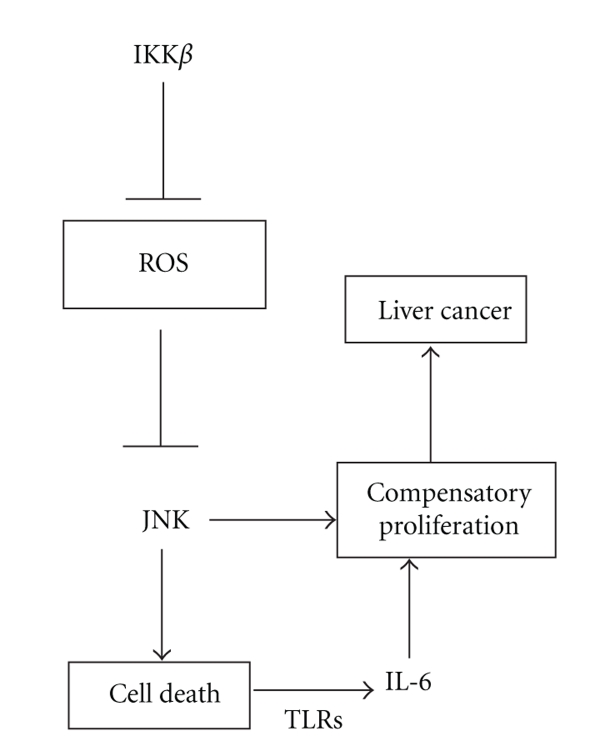


 Based on an NF-*κ*B and JNK study in an animal hepatocarcinogenesis model, regulation of ROS-mediated JNK activation is critical for developing cancer. Recent studies have revealed that ROS production accompanies many signaling events, including receptor signaling, and that ROS play critical roles in determining cell fate as second messengers and modifying various signaling molecules. Sustained JNK activation is induced by ROS by activating upstream MEKKs or inhibiting MKPs [70–72]. In fact, enhanced JNK activation in IKK*β*-deficient mice has been assessed. Hepatocyte-specific *Ikk*β**-knockout mice and *Jnk1*-knockout mice were inter-bred and treated with DEN at 15 days of age. Double-knockout mice developed three-fold fewer tumors compared with hepatocyte-specific *Ikk*β**-knockout mice, and a similar decrease was found in maximal tumor diameters. These results suggest that increased ROS and ROS-mediated JNK activation is a critical regulator for HCC development (Figure 3). Viral proteins including the HBV X and HCV core proteins are capable of inducing ROS accumulation in hepatocytes [73].

TAK1 is one of the MEKKs that activate both the NF-*κ*B and JNK pathways. Hepatocyte-specific Tak1-deficient mice display spontaneous hepatocyte death, compensatory proliferation, inflammatory cell infiltration, and perisinusoidal fibrosis at the age of 1 month. Older mice develop multiple cancer nodules characterized by increased expression of fetal liver genes including alpha-fetoprotein. Cultures of primary hepatocytes deficient in Tak1 exhibit spontaneous cell death, which is further increased in response to TNF-*α*. These results indicate that TAK1 is an essential component of cellular homeostasis in the liver [74].

### 4.5. JNK1 and Therapeutic Target for HCC

The findings implicating a pivotal contribution of JNK in HCC development suggest the use of a JNK inhibitor for treating HCC. The most commonly used JNK inhibitor is SP600125, which shows high efficacy in blocking JNK kinase activity. In animal models, this compound is effective against acute hepatitis induced by acetaminophen or LPS/GalN [51, 75]. In a mouse model, the cell-permeable JIP peptide, which interferes with the interaction between JIP and JNK to inhibit JNK activity, suppresses chemically induced HCC [59]. 

## 5. HCC and TLRs

### 5.1. TLR Signaling

Innate immunity represents the first line of protection against microbial pathogens and is mediated by macrophages and dendritic cells. Although it was initially suggested to be a nonspecific response, innate immunity discriminates a variety of pathogens through the function of pattern-recognition receptors (PRRs) such as TLRs. These receptors recognize microbial components known as pathogen-associated molecular patterns [76]. Thirteen mammalian TLRs have been described; 10 are expressed in humans, and each is responsible for recognizing distinct bacteria, virus, and fungi microbial structures. The two most eagerly studied are TLR2 and TLR4, the PRRs for gram-negative and gram-positive bacterial products, respectively. TLR4 is also the major receptor recognizing endogenous ligands released from damaged or dying cells. TLRs are characterized by two conserved regions: the extracellular leucine-rich region and the cytoplasmic Toll/IL-1 receptor (TIR) domain. A detailed characterization of the TLRs is described in other chapters. 

TLRs share the initial common activation pathway mediated by the TIR domain. After receptor activation, two signaling pathways mainly exist; one is through the adapter protein myeloid differentiation factor 88 (MyD88), and the other is not. All superfamily receptors, with the exception of TLR3, use MyD88 to initiate signaling. In some cases, MyD88 acts in concert with other adaptors, such as MAL/TIRAP, in the response triggered by stimulating TLR4, TLR1/2, and TLR2/6. In contrast, TLR3-mediated signaling requires only the TRIF adaptor molecule, which is also recruited by TLR4 in association with the other adaptor, TRAM [77]. In the MyD88-dependent pathway, MyD88 is associated with IRAK4, IRAK1, and/or IRAK2. IRAK4 in turn phosphorylates IRAK1, and IRAK2 promotes their association with TRAF6, which serves as a platform to recruit TAK1 kinase. Once activated, TAK1 activates the IKK complex and eventually activates NF-*κ*B and JNK. The IRF7 transcription factor is also activated downstream of TLRs 7, 8, and 9, leading to its translocation into the nucleus and to activation of IFN and IFN-inducible genes. TLR3 and TLR4 both signal through the TRIF adaptor, which interacts with TRAF3 to activate the nonclassical IKKs and the IKK, resulting in the dimerization and activation of IRF3, which then translocates to the nucleus activating the transcription of IFN and IFN-induced genes [78].

### 5.2. TLR Signal and Liver Disease

The liver may be exposed to bacteria from the intestine via the portal vein, leading to an uncontrolled innate immune system that may result in inflammatory liver disorders [79]. Many factors are capable of activating TLRs in the liver. Among them, HBV, HCV, alcoholic liver disease, and NASH are important etiologies for HCC. 

The TLR ligands TLR4 and 9 inhibit viral replication in HBV-transgenic mice [80]. In the absence of HBeAg, HBV replication is associated with upregulation of the TLR2 pathway, leading to increased TNF*α* production, demonstrating a potentially important interaction between HBV and the innate immune response [81].

HCV can activate innate immune systems to produce inflammation. The HCV core and NS3 proteins activate TLR2 on monocytes to induce cytokines in a NF-*κ*B- and JNK-dependent manner [82]. The NS3 protein interacts directly with TBK1, resulting in decreased TBK1-IRF3 interaction and inhibition of IRF3 and IFN transcription. The NS3 protein also impedes both IRF3 and NF-*κ*B activation by reducing functional TRIF abundance [83]. Many other *in vitro* studies have been reported, but the *in vivo* condition is still unclear. 

Excessive alcohol intake is associated with increased intestinal permeability and elevated endotoxin levels [84]. LPS activates TLR4 on Kupffer cells and increases proinflammatory cytokine production. Antibiotic treatment reduces the sensitivity of alcoholic liver disease [85, 86].

Intestinal bacteria seem to be important in NASH pathogenesis. In NASH, ob/ob mice exhibit increased hepatic sensitivity to LPS and developed steatohepatitis [87]. In a methionine/choline-deficient NASH model, TLR4-deficient, but not TLR2-deficient mice, exhibited less intrahepatic lipid accumulation [88].

### 5.3. TLR Signal and HCC

All of the diseases described above are associated with the development of HCC. Therefore, it seems clear that TLRs are involved in the development of HCC. It is very important to analyze which cell types are involved in the process to understand liver tumorigenesis. Kupffer cells may be the major cells expressing TLRs in liver. Kupffer cells are resident liver macrophages and express most major TLRs [89]. In contrast, hepatocytes, the liver parenchymal cells, show weak TLR 2 and TLR4 expression and less response against their ligands [90, 91]. TLR2 expression in hepatocytes is upregulated by LPS, TNF, and others, suggesting that hepatocytes become more sensitive in the inflammatory condition [92]. 

Mice deficient in TLR4 and MyD88, but not TLR2, have a marked decrease in the incidence, size, and number of chemically induced (DEN) liver cancer tumors, indicating a strong contribution of TLR signaling to hepatocarcinogenesis [79]. It is assumed that dying hepatocytes following DEN may activate myeloid cells such as Kupffer cells via TLRs and induce proinflammatory cytokines and hepatomitogens, which enhance the development of HCC [93].

 MyD88 is an adapter molecule for TLRs necessary for NF-*κ*B, and MyD88 ablation strongly suppresses DEN-induced hepatocarcinogenesis [32]. IL-6 induction and liver injury are dependent on signaling via MyD88. Collectively, TLR4-MyD88 signaling appears to be essential for hepatocarcinogenesis.

## 6. Conclusion

**Figure 4:**
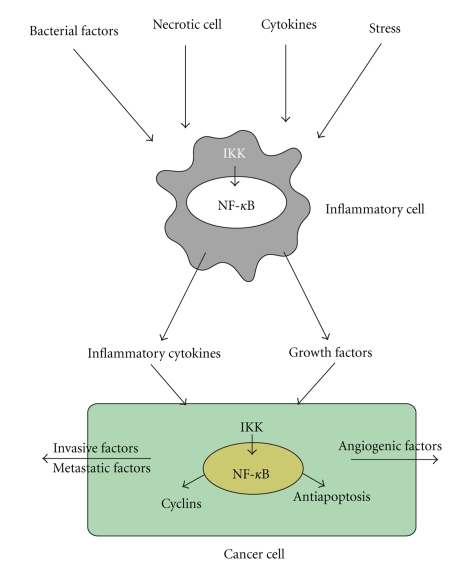


Participation of the IKK*β*/NF-*κ*B and JNK signaling pathways in carcinogenesis differs among organs, cells, and models. The crosstalk between an inflammatory cell and a neoplastic cell, which is instigated by the activation of NF-*κ*B, is critical for tumor organization (Figure 4). JNKs also regulate cell proliferation and apoptosis, making them the main tumor-promoting protein kinases. TLRs play roles in cytokine and hepatomitogen expression mainly in myeloid cells and may promote liver tumorigenesis. These observations suggest that a better understanding of these signaling pathways in the liver will help us understand the mechanism of hepatocarcinogenesis and provide a new therapeutic target for HCC.

## References

[1] Bosch FX, Ribes J, Díaz M, Cléries R (2004). Primary liver cancer: worldwide incidence and trends. *Gastroenterology*.

[2] Koike K, Tsutsumi T, Fujie H, Shintani Y, Moriya K (2002). Molecular mechanism of viral hepatocarcinogenesis. *Oncology*.

[3] Fausto N (1999). Mouse liver tumorigenesis: models, mechanisms, and relevance to human disease. *Seminars in Liver Disease*.

[4] Sakurai T, Maeda S, Chang L, Karin M (2006). Loss of hepatic NF-*κ*B activity enhances chemical hepatocarcinogenesis through sustained c-Jun N-terminal kinase 1 activation. *Proceedings of the National Academy of Sciences of the United States of America*.

[5] Kato N, Yoshida H, Kioko Ono-Nita S (2000). Activation of intracellular signaling by hepatitis B and C viruses: C-viral core is the most potent signal inducer. *Hepatology*.

[6] Caldwell SH, Crespo DM (2004). The spectrum expanded: cryptogenic cirrhosis and the natural history of non-alcoholic fatty liver disease. *Journal of Hepatology*.

[7] Calle EE, Kaaks R (2004). Overweight, obesity and cancer: epidemiological evidence and proposed mechanisms. *Nature Reviews Cancer*.

[8] Park EJ, Lee JH, Yu G-Y (2010). Dietary and genetic obesity promote liver inflammation and tumorigenesis by enhancing IL-6 and TNF expression. *Cell*.

[9] Dejardin E, Droin NM, Delhase M (2002). The lymphotoxin-*β* receptor induces different patterns of gene expression via two NF-*κ*B pathways. *Immunity*.

[10] Ghosh S, Karin M (2002). Missing pieces in the NF-*κ*B puzzle. *Cell*.

[11] Aslund F, Beckwith J (1999). The thioredoxin superfamily: redundancy, specificity, and gray-area genomics. *Journal of Bacteriology*.

[12] Karin M, Cao Y, Greten FR, Li Z-W (2002). NF-*κ*B in cancer: from innocent bystander to major culprit. *Nature Reviews Cancer*.

[13] Kabrun N, Enrietto PJ (1994). The Rel family of proteins in oncogenesis and differentiation. *Seminars in Cancer Biology*.

[14] Hanahan D, Weinberg RA (2000). The hallmarks of cancer. *Cell*.

[15] Szlosarek PW, Balkwill FR (2003). Tumour necrosis factor *α*: a potential target for the therapy of solid tumours. *The Lancet Oncology*.

[16] Guttridge DC, Albanese C, Reuther JY, Pestell RG, Baldwin AS (1999). NF-*κ*B controls cell growth and differentiation through transcriptional regulation of cyclin D1. *Molecular and Cellular Biology*.

[17] Koch KS, Maeda S, He G, Karin M, Leffert HL (2009). Targeted deletion of hepatocyte Ikk*β* confers growth advantages. *Biochemical and Biophysical Research Communications*.

[18] Karin M, Lin A (2002). NF-*κ*B at the crossroads of life and death. *Nature Immunology*.

[19] Müerköster S, Arlt A, Sipos B (2005). Increased expression of the E3-ubiquitin ligase receptor subunit *β*TRCP1 relates to constitutive nuclear factor-*κ*B activation and chemoresistance in pancreatic carcinoma cells. *Cancer Research*.

[20] Cheng Q, Lee HH, Li Y, Parks TP, Cheng G (2000). Upregulation of Bcl-x an Bfl-1 as a potential mechanism of chemoresistance, which can be overcome by NF-*κ*B inhibition. *Oncogene*.

[21] Kunnumakkara AB, Guha S, Krishnan S, Diagaradjane P, Gelovani J, Aggarwal BB (2007). Curcumin potentiates antitumor activity of gemcitabine in an orthotopic model of pancreatic cancer through suppression of proliferation, angiogenesis, and inhibition of nuclear factor-*κ*B-regulated gene products. *Cancer Research*.

[22] Sakamoto K, Maeda S, Hikiba Y (2009). Constitutive NF-*κ*B activation in colorectal carcinoma plays a key role in angiogenesis, promoting tumor growth. *Clinical Cancer Research*.

[23] Egeblad M, Werb Z (2002). New functions for the matrix metalloproteinases in cancer progression. *Nature Reviews Cancer*.

[24] Maeda S, Hikiba Y, Sakamoto K (2009). Ikappa B kinase*β*/nuclear factor-*κ*B activation controls the development of liver metastasis by way of interleukin-6 expression. *Hepatology*.

[25] Tai D-I, Tsai S-L, Chang Y-H (2000). Constitutive activation of nuclear factor *κ*B in hepatocellular carcinoma. *Cancer*.

[26] Jiang R, Xia Y, Li J (2010). High expression levels of IKK*α* and IKK*β* are necessary for the malignant properties of liver cancer. *International Journal of Cancer*.

[27] Li Z-W, Chu W, Hu Y (1999). The IKK*β* subunit of I*κ*B kinase (IKK) is essential for nuclear factor *κ*B activation and prevention of apoptosis. *Journal of Experimental Medicine*.

[28] Maeda S, Chang L, Li Z-W, Luo J-L, Leffert H, Karin M (2003). IKK*β* is required for prevention of apoptosis mediated by cell-bound but not by circulating TNF*α*. *Immunity*.

[29] Maeda S, Kamata H, Luo J-L, Leffert H, Karin M (2005). IKK*β* couples hepatocyte death to cytokine-driven compensatory proliferation that promotes chemical hepatocarcinogenesis. *Cell*.

[30] Tang G, Minemoto Y, Dibling B (2001). Inhibition of JNK activation through NF-*κ*B target genes. *Nature*.

[31] Fausto N (2000). Liver regeneration. *Journal of Hepatology*.

[32] Naugler WE, Sakurai T, Kim S (2007). Gender disparity in liver cancer due to sex differences in MyD88-dependent IL-6 production. *Science*.

[33] He G, Yu G-Y, Temkin V (2010). Hepatocyte IKK*β*/NF-*κ*B inhibits tumor promotion and progression by preventing oxidative stress-driven STAT3 activation. *Cancer Cell*.

[34] Luedde T, Beraza N, Kotsikoris V (2007). Deletion of NEMO/IKK*γ* in liver parenchymal cells causes steatohepatitis and hepatocellular carcinoma. *Cancer Cell*.

[35] Zhao T, Yang L, Sun Q (2007). The NEMO adaptor bridges the nuclear factor-*κ*B and interferon regulatory factor signaling pathways. *Nature Immunology*.

[36] Hayakawa Y, Maeda S, Nakagawa H (2009). Effectiveness of I*κ*B kinase inhibitors in murine colitis-associated tumorigenesis. *Journal of Gastroenterology*.

[37] Karin M, Yamamoto Y, Wang QM (2004). The IKK NF-*κ*B system: a treasure trove for drug development. *Nature Reviews Drug Discovery*.

[38] Dérijard B, Hibi M, Wu I-H (1994). JNK1: a protein kinase stimulated by UV light and Ha-Ras that binds and phosphorylates the c-Jun activation domain. *Cell*.

[39] Kallunki T, Su B, Tsigelny I (1994). JNK2 contains a specificity-determining region responsible for efficient c-Jun binding and phosphorylation. *Genes and Development*.

[40] Mohit AA, Martin JH, Miller CA (1995). p49(3F12) kinase: a novel MAP kinase expressed in a subset of neurons in the human nervous system. *Neuron*.

[41] Wang X, Destrument A, Tournier C (2007). Physiological roles of MKK4 and MKK7: insights from animal models. *Biochimica et Biophysica Acta*.

[42] Gupta S, Barrett T, Whitmarsh AJ (1996). Selective interaction of JNK protein kinase isoforms with transcription factors. *The EMBO Journal*.

[43] Shaulian E, Karin M (2002). AP-1 as a regulator of cell life and death. *Nature Cell Biology*.

[44] Weston CR, Davis RJ (2002). The JNK signal transduction pathway. *Current Opinion in Genetics and Development*.

[45] Sabapathy K, Hu Y, Kallunki T (1999). JNK2 is required for efficient T-cell activation and apoptosis but not for normal lymphocyte development. *Current Biology*.

[46] Sabapathy K, Wagner EF (2004). JNK2: a negative regulator of cellular proliferation. *Cell Cycle*.

[47] Kuan C-Y, Yang DD, Samanta Roy DR, Davis RJ, Rakic P, Flavell RA (1999). The Jnk1 and Jnk2 protein kinases are required for regional specific apoptosis during early brain development. *Neuron*.

[48] Hirosumi J, Tuncman G, Chang L (2002). A central, role for JNK in obesity and insulin resistance. *Nature*.

[49] Tournier C, Hess P, Yang DD (2000). Requirement of JNK for stress-induced activation of the cytochrome c-mediated death pathway. *Science*.

[50] Hanawa N, Shinohara M, Saberi B, Gaarde WA, Han D, Kaplowitz N (2008). Role of JNK translocation to mitochondria leading to inhibition of mitochondria bioenergetics in acetaminophen-induced liver injury. *Journal of Biological Chemistry*.

[51] Nakagawa H, Maeda S, Hikiba Y (2008). Deletion of apoptosis signal-regulating kinase 1 attenuates acetaminophen-induced liver injury by inhibiting c-Jun N-terminal kinase activation. *Gastroenterology*.

[52] Das M, Sabio G, Jiang F, Rincón M, Flavell RA, Davis RJ (2009). Induction of hepatitis by JNK-mediated expression of TNF-*α*. *Cell*.

[53] Samuel VT, Liu Z-X, Qu X (2004). Mechanism of hepatic insulin resistance in non-alcoholic fatty liver disease. *Journal of Biological Chemistry*.

[54] Kodama Y, Kisseleva T, Iwaisako K (2009). c-Jun N-terminal kinase-1 from hematopoietic cells mediates progression from hepatic steatosis to steatohepatitis and fibrosis in mice. *Gastroenterology*.

[55] Tong C, Yin Z, Song Z (2007). c-Jun NH2-terminal kinase 1 plays a critical role in intestinal homeostasis and tumor suppression. *American Journal of Pathology*.

[56] Shibata W, Maeda S, Hikiba Y (2008). c-Jun NH2-terminal kinase 1 is a critical regulator for the development of gastric cancer in mice. *Cancer Research*.

[57] Minden A, Lin A, McMahon M (1994). Differential activation of ERK and JNK mitogen-activated protein kinases by Raf-1 and MEKK. *Science*.

[58] Clark GJ, Westwick JK, Der CJ (1997). p120 GAP modulates Ras activation of jun kinases and transformation. *Journal of Biological Chemistry*.

[59] Hui L, Zatloukal K, Scheuch H, Stepniak E, Wagner EF (2008). Proliferation of human HCC cells and chemically induced mouse liver cancers requires JNK1-dependent p21 downregulation. *Journal of Clinical Investigation*.

[60] Bost F, McKay R, Dean N, Mercola D (1997). The JUN kinase/stress-activated protein kinase pathway is required for epidermal growth factor stimulation of growth of human A549 lung carcinoma cells. *Journal of Biological Chemistry*.

[61] Weston CR, Wong A, Hall JP, Goad MEP, Flavell RA, Davis RJ (2004). The c-Jun NH2-terminal kinase is essential for epidermal growth factor expression during epidermal morphogenesis. *Proceedings of the National Academy of Sciences of the United States of America*.

[62] Guma M, Rius J, Duong-Polk KX, Haddad GG, Lindsey JD, Karin M (2009). Genetic and pharmacological inhibition of JNK ameliorates hypoxia-induced retinopathy through interference with VEGF expression. *Proceedings of the National Academy of Sciences of the United States of America*.

[63] Strieter RM, Polverini PJ, Arenberg DA (1995). Role of C-X-C chemokines as regulators of angiogenesis in lung cancer. *Journal of Leukocyte Biology*.

[64] Yoshida S, Ono M, Shono T (1997). Involvement of interleukin-8, vascular endothelial growth factor, and basic fibroblast growth factor in tumor necrosis factor alpha-dependent angiogenesis. *Molecular and Cellular Biology*.

[65] Chang Q, Zhang Y, Beezhold KJ (2009). Sustained JNK1 activation is associated with altered histone H3 methylations in human liver cancer. *Journal of Hepatology*.

[66] Tarn C, Lee S, Hu Y, Ashendel C, Andrisani OM (2001). Hepatitis B virus X protein differentially activates RAS-RAF-MAPK and JNK pathways in X-transforming versus non-transforming AML12 hepatocytes. *Journal of Biological Chemistry*.

[67] Hibi M, Lin A, Smeal T, Minden A, Karin M (1993). Identification of an oncoprotein- and UV-responsive protein kinase that binds and potentiates the c-Jun activation domain. *Genes and Development*.

[68] Eferl R, Ricci R, Kenner L (2003). Liver tumor development: c-Jun antagonizes the proapoptotic activity of p53. *Cell*.

[69] Angel P, Karin M (1991). The role of Jun, Fos and the AP-1 complex in cell-proliferation and transformation. *Biochimica et Biophysica Acta*.

[70] Matsuzawa A, Ichijo H (2008). Redox control of cell fate by MAP kinase: physiological roles of ASK1-MAP kinase pathway in stress signaling. *Biochimica et Biophysica Acta*.

[71] Chen F, Beezhold K, Castranova V (2009). JNK1, a potential therapeutic target for hepatocellular carcinoma. *Biochimica et Biophysica Acta*.

[72] Kamata H, Honda S-I, Maeda S, Chang L, Hirata H, Karin M (2005). Reactive oxygen species promote TNF*α*-induced death and sustained JNK activation by inhibiting MAP kinase phosphatases. *Cell*.

[73] Okuda M, Li K, Beard MR (2002). Mitochondrial injury, oxidative stress, and antioxidant gene expression are induced by hepatitis C virus core protein. *Gastroenterology*.

[74] Inokuchi S, Aoyama T, Miura K (2010). Disruption of TAK1 in hepatocytes causes hepatic injury, inflammation, fibrosis, and carcinogenesis. *Proceedings of the National Academy of Sciences of the United States of America*.

[75] Takamura M, Matsuda Y, Yamagiwa S (2007). An inhibitor of c-Jun NH2-terminal kinase, SP600125, protects mice from d-galactosamine/lipopolysaccharide-induced hepatic failure by modulating BH3-only proteins. *Life Sciences*.

[76] Takeuchi O, Akira S (2001). Toll-like receptors; their physiological role and signal transduction system. *International Immunopharmacology*.

[77] O’Neill LAJ, Bowie AG (2007). The family of five: TIR-domain-containing adaptors in Toll-like receptor signalling. *Nature Reviews Immunology*.

[78] Akira S, Uematsu S, Takeuchi O (2006). Pathogen recognition and innate immunity. *Cell*.

[79] Seki E, Brenner DA (2008). Toll-like receptors and adaptor molecules in liver disease: update. *Hepatology*.

[80] Isogawa M, Robek MD, Furuichi Y, Chisari FV (2005). Toll-like receptor signaling inhibits hepatitis B virus replication in vivo. *Journal of Virology*.

[81] Visvanathan K, Skinner NA, Thompson AJV (2007). Regulation of Toll-like receptor-2 expression in chronic hepatitis B by the precore protein. *Hepatology*.

[82] Dolganiuc A, Oak S, Kodys K (2004). Hepatitis C core and nonstructural 3 proteins trigger toll-like receptor 2-mediated pathways and inflammatory activation. *Gastroenterology*.

[83] Li K, Foy E, Ferreon JC (2005). Immune evasion by hepatitis C virus NS3/4A protease-mediated cleavage of the Toll-like receptor 3 adaptor protein TRIF. *Proceedings of the National Academy of Sciences of the United States of America*.

[84] Bjarnason I, Ward K, Peters TJ (1984). The leaky gut of alcoholism: possible route of entry for toxic compounds. *The Lancet*.

[85] Adachi Y, Moore LE, Bradford BU, Gao W, Thurman RG (1995). Antibiotics prevent liver injury in rats following long-term exposure to ethanol. *Gastroenterology*.

[86] Uesugi T, Froh M, Arteel GE, Bradford BU, Thurman RG (2001). Toll-like receptor 4 is involved in the mechanism of early alcohol-induced liver injury in mice. *Hepatology*.

[87] Yang SQ, Lin HZ, Lane MD, Clemens M, Diehl AM (1997). Obesity increases sensitivity to endotoxin liver injury: implications for the pathogenesis of steatohepatitis. *Proceedings of the National Academy of Sciences of the United States of America*.

[88] Rivera CA, Adegboyega P, van Rooijen N, Tagalicud A, Allman M, Wallace M (2007). Toll-like receptor-4 signaling and Kupffer cells play pivotal roles in the pathogenesis of non-alcoholic steatohepatitis. *Journal of Hepatology*.

[89] Schwabe RF, Seki E, Brenner DA (2006). Toll-Like receptor signaling in the liver. *Gastroenterology*.

[90] Matsumura T, Degawa T, Takii T (2003). TRAF6-NF-*κ*B pathway is essential for interleukin-1-induced TLR2 expression and its functional response to TLR2 ligand in murine hepatocytes. *Immunology*.

[91] Liu S, Gallo DJ, Green AM (2002). Role of toll-like receptors in changes in gene expression and NF-*κ*B activation in mouse hepatocytes stimulated with lipopolysaccharide. *Infection and Immunity*.

[92] Matsumura T, Ito A, Takii T, Hayashi H, Onozaki K (2000). Endotoxin and cytokine regulation of toll-like receptor (TLR) 2 and TLR4 gene expression in murine liver and hepatocytes. *Journal of Interferon and Cytokine Research*.

[93] Maeda S, Omata M (2008). Inflammation and cancer: role of nuclear factor-kappaB activation. *Cancer Science*.

